# Criminal Abortion

**Published:** 1884-08-20

**Authors:** 


### CRIMINAL ABORTION.

"It is well known by the practicing physicians of Atlanta that many young women in this city, and some who have come from other surrounding States, have been unfortunate, as the phrase goes, in receiving deposits which have not passed current subsequently, and that extraordinary means have been resorted to for the removal of the deposits. How far the bright escutcheon of the medical profession has been tarnished by aiding and abetting this financial transaction must be decided in the solution of the allegation that every man has his price. That some have borne their crest so loftily as to refuse compliance with the appeals of family influence, and to resist all the temptations of a golden harvest, is in striking contrast with others who have lent a willing hand to screen the victim for the miserable consideration of a few dollars."

We feel that the above extract from the Atlanta Medical and Surgical Journal, relative to criminal abortion in Atlanta, demands a rebuttal.

While no one has a greater contempt and hatred for criminal abortionists than the editors of this Journal, yet we feel that it is due to the honor of the Profession, not less than to the virtue of our women, to say that the statement is largely overdrawn, and is, therefore, unjust and offensive to our people.

That there are occasional instances of the kind referred to in Atlanta, as in all other places of any considerable size, no one will deny; but that the proportion of such cases is larger than elsewhere we do not believe there is good ground for asserting.

In reference to the allegation that every man has his price, we are told "That some have borne their crest so loftily as to refuse compliance," etc., leaving the inference that many others have not done so.

To this we reply that we do not believe that any true and legitimate graduate of medicine in this city is an abortionist, nor that the honor and moral standing of the Profession here is in any respect below that of other places, and we feel that the above sweeping and offensive charge will excite not only the astonishment, but the indignation of every true man of the Profession resident in our city.

